# Inter-individual Variability in Soccer Players of Different Age Groups Playing Different Positions

**DOI:** 10.2478/hukin-2014-0023

**Published:** 2014-04-09

**Authors:** Pantelis Nikolaidis, Gal Ziv, Ronnie Lidor, Michal Arnon

**Affiliations:** 1 Exercise Physiology Laboratory, Nikea, Attiki, Greece.; 2 The Zinman College of Physical Education and Sport Sciences, Wingate Institute, Israel.; 3 Faculty of Education, University of Haifa, Israel.

**Keywords:** exercise, performance, talent development, anthropometric and physiological characteristics

## Abstract

The purpose of this study was twofold: (a) to profile physical characteristics and motor abilities of three age groups of soccer players – under 14 years, 14–17, and over 17, playing different positions – goalkeepers, defenders, midfielders, and forwards; and (b) to examine the inter-individual variability among the players in each age group in all physical and physiological measurements performed in the study. In addition, anthropometric, power, strength, and flexibility tests were administered. Findings showed large inter-individual variability in all three age groups and in all playing positions. Differences between playing positions were found only in the 14–17 group (body mass) and in the over-17 group (body height, body mass, fat-free mass, and mean power in the Wingate Anaerobic Test). Due to the observed large inter-individual variability, it was concluded that the findings obtained in the physical and physiological tests should be interpreted with caution when attempting to differentiate between successful and unsuccessful soccer players, as well as when trying to predict future success in soccer.

## Introduction

Physical characteristics and motor abilities of female and male elite players who play different ball games have been reviewed in recent years. The research covering this topic has been conducted for example in basketball (Ziv and Lidor, 2009), handball (Lidor and Ziv, 2011), and volleyball (Lidor and Ziv, 2010). One of the main purposes of these reviews was to identify those variables among players that can be effectively used by coaches to predict future success of athletes in these sports. One of the desired objectives of any training program is to develop talent in youth athletes and to recruit those with the highest potential to reach the championship level in a given sport.

In the aforementioned reviews, as well as in other studies examining which variables can be used to predict future ability in sport, relevant information on different types of variables was obtained. For example, physical and physiological variables were examined in a number of studies (e.g., Gil et al., 2007; Hoare, 2000; Reilly et al., 2000a), while in others psychological variables were assessed (e.g., Morris, 2000; Van Yperen, 2009). In some studies, both physiological and psychological variables were measured (e.g., Reilly et al., 2000b). It is worth noting that no clear-cut findings were observed in these studies as to which variables can be used to predict future success in sport, and therefore, it was concluded that the ability to make such predictions is limited (Lidor et al., 2009). Indeed, success in sport is a complex arrangement of a wide array of variables, among them physical, physiological, psychological, and social; various combinations of them can lead to the achievement of a high level of proficiency. For example, one athlete can have average physiological capacities, yet he or she exhibits a high level of motivation. Another athlete may demonstrate superior physiological capabilities, but with a moderate level of motivation. Both athletes can be successful in a certain sport, however the success is due to different individual variables.

Typically, in both the reviews and studies examining which variables can be used to predict future performance in sport, only means and standard deviations of the measured variables were reported. This is to say that the inter-individual variability among the athletes was not discussed. Indeed, means and standard deviations are the most common statistical values to be used when (a) attempting to profile performance variables of athletes, and (b) aiming to compare different athletes with different profiles, such as athletes of different ages, of varied skill levels, with varying amount of experience in competitive sports, or playing different positions in a given sports discipline.

Unfortunately, data on inter-individual variability in sports are scarce. In one study of pistol shooting (Ball et al., 2003), errors in body sway, aim-point fluctuation, and performance were found to be individual-specific. The authors of this study concluded that individual analyses should be prioritized when examining elite sport performances. In a recent study on a group of female volleyball players (Nikolaidis et al., 2012), large inter-individual variability was reported in most of the physical characteristics (e.g., body mass, body height) and physiological variables (e.g., aerobic capacity, anaerobic power) measured in the players. In addition, inter-individual variability was also observed in all age groups classified in this volleyball study: under 14, 14–18, and over 18 years of age. Individual differences were also demonstrated in two studies on soccer, one in which physical and physiological characteristics of top-level soccer players were examined (Reilly et al., 2000a), and the other where blood-lactate levels were assessed in 28 English Division One Clubs (2^nd^ highest league) players performing soccer drills (Little and Williams, 2007). In another soccer study (Hencken and White, 2006), despite the homogeneity in a squad of 24 professional soccer players from a Premiership Football Club in England, an examination of within-position data revealed inter-individual variability. For example, high variation in body mass was found in the midfielders.

Examples of inter-individual variability have also been observed in other fields. In one sport psychology study of elite golfers (Hassmen et al., 2004), state anxiety and self-confidence were examined. The participants were 10 members of the Swedish National Amateur Golf Team. The golfers were observed by the researchers during one competitive season. The results of this study showed that inter-individual variability in pre-competition anxiety was considerable among the golfers. Lastly, in one study on running, inter-individual variability was also shown in the oxygen cost of running in well-trained distance runners (±7.1% in male runners and ±5.2% in female runners) (Helgerud et al., 2010).

In order to develop effective training programs that reflect the specific needs of individual athletes, as well as to use the appropriate variables when attempting to predict future success in sport, it may be important to also examine the inter-individual variability that exists among athletes. This information can be used effectively by sport coaches as well as strength and conditioning coaches to enable an individual athlete to gain the greatest benefit from his or her training program. In soccer, for example, it has already been argued that heterogeneity exists in the anthropometric characteristics and physiological variables of players in elite teams, and that various factors can predispose players’ success (Reilly et al., 2000a). An interesting question is whether these arguments are valid for soccer players of different age groups playing different positions.

Therefore, the purpose of this study was twofold: (a) to profile physical characteristics and physiological variables of three age groups of soccer players – under 14 years, 14–17, and over 17, playing different positions – goalkeepers, defenders, midfielders, and forwards; and (b) to examine the inter-individual variability among the players in each age group in all physical and physiological measurements performed in the study. Specifically, our objective in the current study was to examine differences and inter-individual variability in power and strength attributes in soccer players of different age groups playing different positions. These two attributes – power and strength – have been found to be essential for achieving a high level of proficiency in this game (Ziv and Lidor, 2011). In addition, we also wanted to assess these attributes specifically in goalkeepers, since there is very little data on players at this position. For example, in a review of studies on soccer goalkeepers (Ziv and Lidor, 2011), only four studies examining power and strength values in adult goalkeepers were found.

## Material and Methods

### Participants

Two hundred and forty-nine male soccer players participated in the current study. The players were divided into three age groups: (a) age 14 and under (n = 20; mean age = 13.23±.52 years), (b) from ages 14 to 17 (n = 94; mean age = 15.47±.83 years), and (c) over the age of 17 (n = 135; mean age = 20.45±3.48 years). The players were members of B, C, D, and E Series clubs (2^nd^, 3^rd^, 4^th^, and 5^th^ best leagues in soccer, respectively) in Greece. The rationale for the selection of these age classifications for the players participating in the study was that national and international soccer competitive leagues and tournaments are typically organized according to these age groups.

Soccer experience of the players was 4.31±2.27 years for the under-14 group, 6.18±2.78 years for the 14–17 group, and 10.20±5.09 years for the over-17 group. Total weekly training time was 261.18±93.10 min·wk^−1^ for the under-14 group, 345.61±105.10 min·wk^−1^ for the 14–17 group, and 350.14±140.94 min·wk^−1^ for the over-17 group. All players completed an informed consent form prior to participation. The parents of the players who were under the age of 18 approved the participation of their children in the study. In addition, the study was approved by the Ethics Committee of the Zinman College of Physical Education and Sport Sciences.

### Procedure

Upon arrival at the Exercise Physiology Laboratory, participants were provided with a verbal explanation of the experimental design of the study. All testing was conducted by an experienced exercise physiologist. Testing took place during the competitive phase of the 2008–9, 2009–10, and 2010–11 seasons, on weekdays between 8:00 a.m. and 2:00 p.m. The testing order was similar for all groups: the physical characteristics were measured first, followed by measurement of the physiological variables. Testing lasted about 90 min for each player. To prevent dehydration, ad libitum drinking was permitted for the players after the completion of the jumping tests. All participants performed a standardized warm-up, which included 10 min of cycling and five min of stretching.

### Testing Protocol

A series of physical and physiological tests was administered to the three groups of players. Testing devices and protocols are presented separately for the physical characteristics and physiological variables, according to the order of testing.

### Physical characteristics

Body height and body mass were measured using a stadiometer (SECA, Leicester, UK) and an electronic scale (HD-351, Tanita, Illinois, USA), respectively. Percent of body fat was calculated from the sum of 10 skinfolds using a skinfold caliper (Harpenden, West Sussex, UK). Calculations were based on the formula proposed by Parizkova (1978). Three trials were given for each anthropometric measurement in rotational order, and the average value was recorded.

### Physiological and fitness tests

The players underwent seven tests, as follows:

- A sit-and-reach test for measuring flexibility – players completed the test twice, and the better score was recorded. A 15-cm advantage was given, namely that reaching the toes resulted in a score of 15 cm, reaching five cm further than the toes resulted in a score of 20 cm, and reaching five cm above the toes resulted in a score of 10 cm. The 15-cm advantage was provided in order to assess the performance of those who could not reach their toes. This test was found to have high test-retest reliability [intra-class correlation coefficient (ICC) > .98)] (Gabbe et al., 2004);
- A PWC_170_ test for predicting maximal work capacity – power work capacity at a heart rate (HR) of 170 beats per minute (PWC_170_) was employed as a measure of cardiorespiratory power (Astrand et al., 2003). The players were asked to cycle on a cycle ergometer (Monark Ergomedics 828, Monark, Sweden) for two 3-min sessions against an incremental work load. HR was recorded at the end of each session. The findings were plotted on a workload-HR graphical display, and PWC_170_ was calculated based on the linear relationship between work load and HR. The test-retest reliability of this test was found to be moderate to high (*r* = .76) (Borg and Dahlstrom, 1962);
- A *vertical jump* (VJ) test for measuring the explosive power of the legs – players completed two counter-movement jumps, with arm swing allowed, using the photoelectric cells system Optojump (Microgate, Bolzano, Italy). The higher jump was recorded. Various VJ protocols were found to be highly reliable (*r* > .97) (Aragón-Vargas, 2000);
- An isometric handgrip strength test – players were asked to sit with the elbow of their testing arm resting on a table and bent at approximately 90°. They were then instructed to squeeze the handle of a handgrip dynamometer (Takei, Tokyo, Japan) as strongly as possible for 5 s. Two trials were performed for each hand, with a 1-min rest interval between the trials. The better of the trials was recorded as the maximal effort for each hand (Skinner, 2005). In one study, the test-retest reliability of this test was found to be high (*ICC* = .98) (Essendrop et al., 2001);
- Isometric back and leg strength tests – two tests were performed. In the first one (combined back-and-leg test), the chain length on the dynamometer was adjusted so that the players squatted over the dynamometer with their knees flexed at approximately 30°. In the second test (back strength test), their legs were straight and their back was flexed to allow the bar to be at the level of the patella (Skinner, 2005). A back dynamometer (Takei, Tokyo, Japan) was used in the two tests. In one study, the isometric back flexion and extension test was found to be reliable (*ICC* = .93–.97) (Essendrop et al., 2001).
- A force-velocity (F-v) test – this test was performed for the legs on a cycle ergometer (Monark Ergomedics 874, Monark, Sweden). This test employed various applied braking forces that elicit different pedaling velocities in order to derive maximal power (Heller, 2005). The players performed five supramaximal pedal sprints, each lasting 7 s, against an incremental braking force. The test began with a braking force of 19.6 N for arm cranking and 29.4 N for the cycle ergometer. In every subsequent sprint, 9.8 N was added. The recovery period between each exercise bout lasted five min. During each sprint, participants were verbally encouraged to reach their maximal velocity as quickly as possible. The value of peak velocity was recorded in each sprint and used to estimate F-v parameters. Based on the inverse linear F-v relationship, theoretical maximal values of force (F_0_) and velocity (*v_0_*) were calculated, and maximal power (P_max_) was calculated from the equation: P_max_ = 0.25 F_0_ v_0_;
- The Wingate Anaerobic Test (WAnT) – from a stationary position, participants were asked to pedal as hard as they could for 30 s on a cycle ergometer (Monark Ergomedics 874, Monark, Sweden), with a resistance equal to 7.5% of their body mass. Mechatronic hardware recorded each revolution, and specialized software (Papadopoulos and Nikolaidis, Athens, Greece) calculated peak and mean power output. This test of anaerobic power has been found to be both valid and reliable (Inbar et al., 1996);

### Statistical Analyses

Descriptive statistics are presented as means±*SD*. A one-way analysis of variance (ANOVA) was used to examine differences between positions in each age group on each of the 18 physical and physiological tests. In order to control for multiple comparisons, a Bonferroni correction was used and an alpha level of .003 was set for all significance testing. When statistically significant differences were found, a Tukey’s HSD post-hoc procedure was performed. The Levene’s test of homogeneity of variance was used to assess whether variance differed between the three age groups.

Z-scores for two players from each age group for each playing position were calculated based on both physical and physiological tests. The Z-scores were calculated for four categories: (a) anthropometrics, (b) power, (c) strength, and (d) flexibility. The reliability of these categories was assessed using Cronbach’s Alpha. The two players who were selected from each age group exhibited similar Z-scores.

## Results

Results are presented separately for the inter-individual variability, physical characteristics and physiological variables.

### Inter-individual Variability

The Levene test for equality of variance indicated that no statistically significant differences in variance were found between the age groups in most physical and physiological measurements. The only differences found were between a number of age-related physical characteristics (i.e., body size) of the under-14 group and over-17 group. A large individual variability exists in most of the variables in all the three age groups for each playing position. In some cases (body height, body mass), it appears that individual variability decreases as age increases. These findings are in line with the results of the Levene test for homogeneity of variance, as mentioned previously. In most cases, variability is large in all age groups.

**Figure 1.**
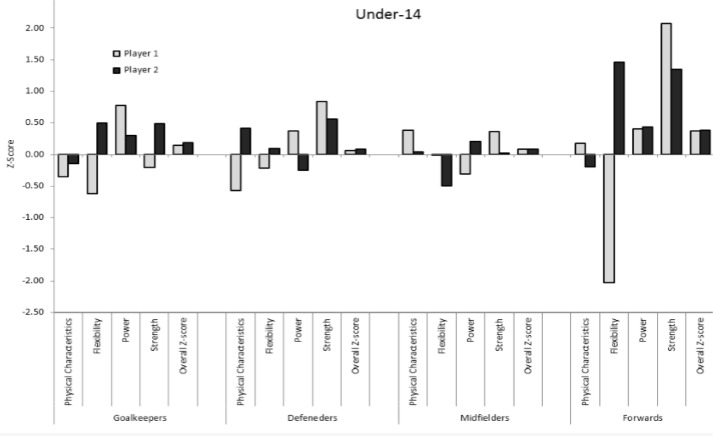
The overall Z-scores and the different Z-scores of two soccer players from each age group and the inter-individual variability within each category of the soccer players in the under-14 group.

**Figure 2.**
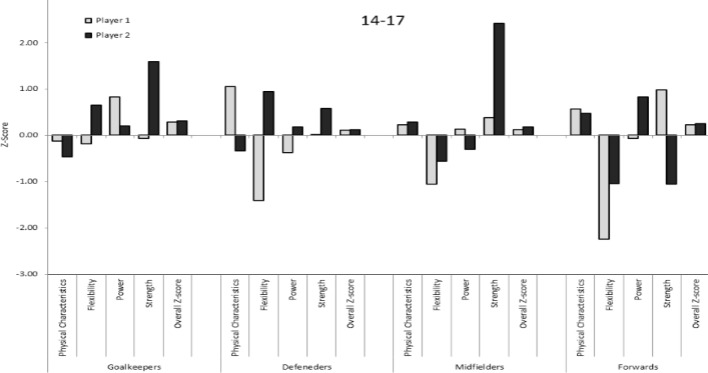
The overall Z-scores and the different Z-scores of two soccer players from each age group and the inter-individual variability within each category of the soccer players in the 14–17 group.

**Figure 3.**
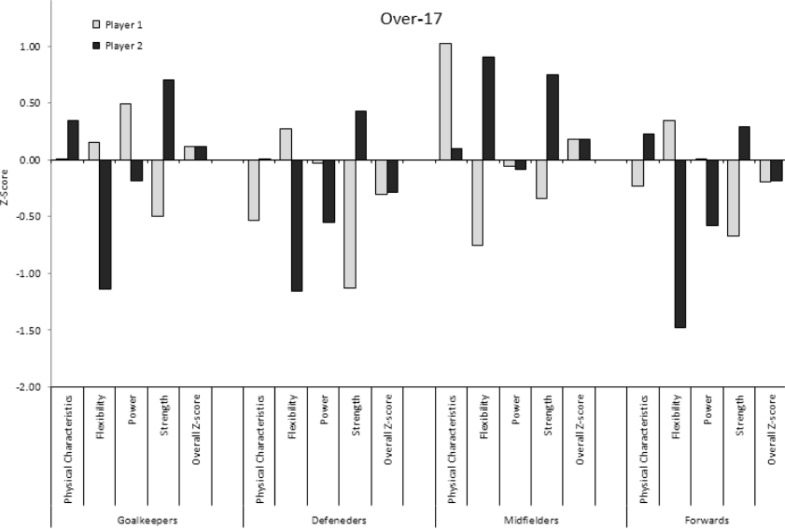
The overall Z-scores and the different Z-scores of two soccer players from each age group and the inter-individual variability within each category of the soccer players in the over-17 group.

The overall Z-scores and the different Z-scores of two soccer players in each playing position from each age group, and the inter-individual variability within each category (physical characteristics, flexibility, power, and strength) are presented in Figures 1, 2, and 3 for the under-14, 14–17, and over-17 players, respectively. While the overall Z-scores of the two players in each playing position are similar, the Z-scores of the individual categories vary greatly. For example, two defenders in the 14–17 age-group have an overall Z-score of approximately 0.2. However, the Z-score for flexibility is −1.5 for one player and 1.0 for the second player. In contrast, the Z-score for anthropometrics is 1.2 for one player and −0.3 for the second player. Another example of two midfielders in the over-17 group shows an overall Z-score of approximately 0.3 for the two players. However, while one player has a Z-score of −0.8 in flexibility, the second player has a Z-score of 0.9 – a difference of almost 2 *SDs*. These two players also show a difference of approximately 1 *SD* in anthropometrics and strength. As can be seen in Figures 1, 2, and 3 large differences in the individual category Z-scores can be observed in players with similar overall Z-scores.

### Physical Characteristics and Motor Abilities

Descriptive statistics of all tests for the under-14, 14–17, and over-17 groups are presented in Tables 1, 2, and 3, respectively. Using an alpha value of .003 after correcting for multiple comparisons, only body mass differences were found to be significant in the 14–17 group (Table 2). In addition, only four variables were found to be significant in the over-17 group: body height, body mass, FFM, and mean power in the WAnT (Table 3).

## Discussion

The main finding of this study was that inter-individual variability exists in most physical and physiological tests in all playing positions in soccer: goalkeepers, defenders, midfielders, and forwards, in each of the three age groups considered (under-14, 14–17, and over-17). In addition, it was found that players playing the same position who had similar overall Z-scores arrived at these scores with different individual Z-scores in both the physical and physiological tests.

### Inter-individual Variability

The existence of a large inter-individual variability as found in the current study is of importance for both researchers and practitioners. For researchers, a cautious approach should be adopted when concluding that certain physical characteristics or physiological variables are related to improved performance. It is proposed that future observational studies comparing achievements of different groups of athletes (e.g., elite vs. near-elite athletes, athletes of different skill levels, players playing different positions in a given sport) should include data on the players’ inter-individual variability. Analyses of the inter-individual variability will help researchers increase their understanding of the actual/practical differences that exist between athletes.

The presence of inter-individual variability should instill caution in practitioners when assessing the ability of athletes to succeed in a certain sport. Success in sport is based on many variables (i.e., physical, physiological, psychological, and/or social), and practitioners should look at the individual player from a holistic viewpoint rather than a reductionist one. Indeed, currently there is no clear evidence that skill tests have predictive value in either talent detection or development in sport (Lidor et al., 2009). **It has already been found that among top soccer teams, heterogeneity in physical skills and motor abilities exists, and therefore it is not possible to isolate individual prerequisites for success with a high level of confidence (Reilly et al., 2000a).**

The fact that at least in some players a similar overall Z-score was based on very different skill-specific Z-scores provides support for the abovementioned point – namely, that there are several means for reaching a similar end. One player can have above-average power values but below-average anthropometric values, and vice versa. However, both of these players can show a similar overall performance.

It should be noted that the tests implemented in the current study measured power, strength, anthropometry, and flexibility. Other attributes, such as aerobic capacity, agility, and speed, were not measured. Although studies of individual differences in such attributes are needed, we already believe that inter-individual variability exists, and that such variability is not limited to soccer. Indeed, similar inter-individual variability was found in a study of physical characteristics and physiological attributes of female volleyball players (Nikolaidis et al., 2012).

While most variables showed large inter-individual variability, some did not. More specifically, the inter-individual variability of body height and body mass decreased as the age increased. This is not surprising, due to the fact that inter-individual variability in skeletal maturation can be high in young athletes (Malina et al., 2007). When the process of maturation ends, it is likely that the inter-individual variability of anthropometric characteristics in a rather homogenous group of athletes would decrease.

### Physical Characteristics and Motor Abilities

The reported values of the physical tests in the current study tended to be similar, or lower, than those found in previous studies. For example, in a study of soccer players between the ages of 14–21 years, body height values ranged from 174 to 180 cm, and body mass values ranged from 63 to 74 kg (Gil et al., 2007). These values are similar to those found in players aged 14–17 and the over-17 players in the current study (body height: 172–182 cm; body mass: 62–81 kg). Similar body height and body mass values were also found in a group of under-18 and under-16 players (mean body height: 176 cm and 171 cm, respectively; mean body mass: 63 kg and 58 kg, respectively) (Leatt et al., 1987). Body height and body mass values in the current study were also similar to those reported for internal-level soccer players (body height: 172–184 cm; body mass: 68–79 kg) (Franks et al., 1999). Lastly, the findings for body height and body mass in the under-14 players in the current study were similar to those found in a group of under-14 players from Hong Kong (Wong et al., 2009).

VJ values measured in the current study (approximately 36–42 cm) were similar, although somewhat lower, when compared to those of players aged 14–21 years (approximately 40–44 cm) (Gil et al., 2007), and to those of adult players playing in Divisions 1 and 2 in France (39–41 cm) (Cometti et al., 2001).

In the current study, the only differences between players playing different positions were found in body mass for the 14–17 group, and for body height, body mass, FFM, and WAnT mean power in the over-17 group. Previous results showed that anaerobic power values were similar between players playing different positions (Arnason et al., 2004; Davis et al., 1992; Gil et al., 2007). In addition, goalkeepers were found to be heavier than field players in previous investigations (Arnason et al., 2004; Davis et al., 1992; Gil et al., 2007), similar to the findings of the current study.

In the under-14 group, after adjusting for multiple comparisons, no statistically significant differences were found between players playing different positions in any of the tests. However, forwards tended to have slightly lower body height and body mass than players playing other positions (Table 1). Similarly, in a group of under-14 players from Hong Kong, body height and body mass were slightly lower in forwards than in players of other positions (Wong et al., 2009).

## Conclusion

The data from the current study suggest that large inter-individual variability can be observed in both adolescent and adult soccer players. This variability is present within each playing position. Soccer coaches as well as strength and conditioning coaches should be aware of this variability, and therefore interpret their players’ achievements in the physical and physiological tests with caution. We recommend adopting a holistic approach for predicting future success in soccer. When possible, it is suggested that researchers report on the inter-individual variability in their studies dealing with highly skilled athletes.

## Figures and Tables

**Table 1:** Descriptive statistics of the applied tests of the aged under-14 players (means±SD).

Test	Under-14 Group (*n* = 20)			
	Goalkeepers (*n* = 3)	Defenders (*n* = 5)	Midfielders (*n* = 7)	Forwards (*n* = 5)
*Anthropometrics*				
Body height (cm)	169.70 ± 5.83	165.94 ± 14.54	163.63 ± 8.67	158.06 ± 13.08
Body mass (kg)	51.13 ± 10.89	49.76 ± 11.68	48.81 ± 11.28	44.83 ± 10.46
BMI (kg·m^−2^)	20.14 ± 2.70	19.80 ± 2.30	20.77 ± 2.32	18.87 ± 1.34
Percent Fat	17.03 ± 5.35	14.78 ± 3.53	15.70 ± 2.83	13.50 ± 1.67
FM (kg)	10.07 ± 4.28	8.34 ± 3.55	8.86 ± 2.46	6.40 ± 1.35
FFM (kg)	47.93 ± 5.38	47.27 ± 12.08	46.79 ± 5.40	41.42 ± 10.10
*PWC_170_*				
Absolute power (W)	138.83 ± 33.62	135.52 ± 41.69	147.04 ± 55.83	134.85 ± 28.50
Relative power (W·kg^−1^)	2.44 ± .69	2.43 ± .30	2.65 ± .95	2.91 ± .78
*WanT*				
Peak power (W)	576.13 ± 89.19	537.93 ± 189.06	532.47 ± 129.03	460.02 ± 143.79
Relative peak power (W·kg^−1^)	9.95 ± .88	9.49 ± 1.08	9.45 ± 1.55	9.52 ± .93
Mean power (W)	444.77 ± 75.10	450.79 ± 163.21	433.65 ± 97.41	367.50 ± 111.90
Relative mean power (W·kg^−1^)	7.68 ± .91	7.94 ± 1.06	7.71 ± 1.00	7.61 ± .68
*Force-velocity test*				
Absolute power (W)	645.89 ± 112.41	782.77 ± 273.90	790.56 ± 144.09	615.35 ± 174.56
Relative power (W·kg^−1^)	11.30 ± 2.48	13.88 ± 1.79	14.28 ± 2.25	12.94 ± 2.41
*Vertical jump (cm)*	26.71 ± 7.84	27.57 ± 6.99	29.08 ± 7.60	28.84 ± 6.83
*Isometric strength*				
Right hand grip (kg)	35.37 ± 7.70	30.06 ± 7.07	28.76 ± 3.77	28.02 ± 8.56
Left hand grip (kg)	33.73 ± 6.34	29.24 ± 7.12	27.41 ± 3.55	26.78 ± 6.66
Trunk (kg)	74.67 ± 9.25	91.30 ± 29.78	76.43 ± 20.29	74.80 ± 31.01
Trunk /Legs (kg)	102.67 ± 17.11	111.40 ± 41.75	87.79 ± 28.56	88.00 ± 40.18
*Flexibility*				
Sit and reach (cm)	19.75 ± 6.63	19.35 ± 8.72	20.25 ± 4.00	14.65 ± 5.61

**Table 2:** Descriptive statistics of the applied tests of the aged 14–17 players (means±SD).

Test	14–17 Group (*n* = 94)			
	Goalkeepers (*n* = 8)	Defenders (*n* = 37	Midfielders (*n* = 34)	Forwards (*n* = 15)
*Anthropometrics*				
Body height (cm)	173.04 ± 4.52	172.72 ± 7.63		171.81 ± 7.24
Body mass (kg)	73.54 ± 13.61^**^	65.31 ± 8.10^*^	62.34 ± 9.50	62.71 ± 8.46
BMI (kg·m^−2^)	25.31 ± 6.50	22.19 ± 2.38	20.99 ± 2.78	22.15 ± 1.54
Percent Fat	19.25 ± 6.84	16.70 ± 4.47	14.99 ± 3.06	16.69 ± 2.95
FM (kg)	15.61 ± 9.73	11.25 ± 3.91	9.52 ± 3.41	10.97 ± 2.40
FFM (kg)	60.06 ± 9.00	55.07 ± 6.74	52.61 ± 6.42	54.62 ± 6.81
*PWC_170_*				
Absolute power (W)	141.20 ± 26.97	161.64 ± 32.82	160.06 ± 40.55	157.69 ± 41.02
Relative power (W·kg^−1^)	1.92 ± .39	2.45 ± .45	2.61 ± .72	2.40 ± .48
*WanT*				
Peak power (W)	772.55 ± 140.38	686.54 ± 115.74	666.51 ± 123.88	723.85 ± 129.37
Relative peak power (W·kg^−1^)	10.47 ± 1.78	10.35 ± .95	10.69 ± .70	10.98 ± .88
Mean power (W)	569.04 ± 104.16	542.93 ± 98.11	535.44 ± 82.50	569.76 ± 94.78
Relative mean power (W·kg^−1^)	7.72 ± 1.40	8.19 ± .92	8.63 ± .52	8.65 ± .60
*Force-velocity test*				
Absolute power (W)	952.26 ± 133.84	922.84 ± 246.60	889.54 ± 200.24	983.60 ± 186.77
Relative power (W·kg^−1^)	13.12 ± 3.12	13.87 ± 2.76	14.33 ± 2.65	15.04 ± 2.46
*Vertical jump (cm)*	35.81 ± 7.52	37.88 ± 6.90	37.11 ± 6.66	41.25 ± 5.38
*Isometric strength*				
Right hand grip (kg)	41.35 ± 9.00	38.76 ± 7.50	38.39 ± 8.73	41.86 ± 10.41
Left hand grip (kg)	38.06 ± 9.20	35.75 ± 6.50	37.19 ± 8.93	39.80 ± 8.50
Trunk (kg)	98.63 ± 17.38	106.88 ± 27.59	110.15 ± 22.47	103.17 ± 34.66
Trunk /Legs (kg)	120.38 ± 23.21	129.20 ± 32.01	135.41 ± 28.09	126.93 ± 28.75
*Flexibility*				
Sit and reach (cm)	23.81 ± 5.90	21.19 ± 7.89	20.39 ± 7.60	22.83 7.62

* Significant difference between defenders and midfielders.

** Significant difference between goalkeepers and all other positions.

**Table 3:** Descriptive statistics of the applied tests of the aged over-17 players (means±SD).

Test	Over-17 Group (*n* = 135)			
	Goalkeepers (*n* = 15)	Defenders (*n* = 50)	Midfielders (*n* = 53)	Forwards (*n* = 17)
*Anthropometrics*				
Body height (cm)	181.49 ± 4.75^*^	176.85 ± 5.62	175.20 ± 6.63^#^	178.70 ± 6.13
Body mass (kg)	81.61 ± 9.45^**^	72.43 ± 8.33	71.23 ± 6.45	74.12 ± 9.17
BMI (kg·m^−2^)	24.44 ± 1.71	22.89 ± 2.11	22.83 ± 1.78	23.65 ± 2.18
Percent Fat	16.93 ± 3.14	15.04 ± 3.03	15.15 ± 3.03	14.88 ± 3.39
FM (kg)	13.72 ± 3.22	10.93 ± 3.25	10.70 ± 2.72	11.42 ± 3.72
FM (kg)	66.94 ± 6.31^*^	60.78 ± 6.15	59.33 ± 4.52	64.27 ± 6.95^##^
*PWC_170_*				
Absolute power (W)	218.39 ± 48.69	205.04 ± 45.47	204.20 ± 42.31	196.08 ± 36.22
Relative power (W·kg^−1^)	2.70 ± .51	2.86 ± .53	2.91 ± .53	2.60 ± .41
*WanT*				
Peak power (W)	888.53 ± 108.09	808.68 ± 114.57	807.13 ± 101.77	851.33 ± 133.61
Relative peak power (W·kg^−1^)	11.00 ± .62	11.27 ± .94	11.50 ± .79	11.24 ± 1.00
Mean power (W)	656.68 ± 71.95	626.95 ± 75.74	637.25 ±72.68	677.98 ± 105.24
Relative mean power (W·kg^−1^)	8.16 ± .71^**^	8.77 ± .76^$^	9.09 ± .55	8.96 ± .87
*Force-velocity test*				
Absolute power (W)	1135.71 ± 209.24	1080.52 ± 210.61	1076.27 ± 247.21	1075.15 ± 212.61
Relative power (W·kg^−1^)	14.09 ± 2.30	15.08 ± 2.41	15.33 ± 2.96	14.21 ± 2.31
*Vertical jump (cm)*	37.40 ± 6.87	40.71 ± 7.12	41.52 ± 7.23	43.18 ± 6.39
*Isometric strength*				
Right hand grip (kg)	50.75 ± 5.28	46.88 ± 7.69	46.97 ± 6.89	46.83 ± 9.09
Left hand grip (kg)	48.31 ± 6.47	45.69 ± 8.27	44.46 ± 7.15	45.33 ± 8.41
Trunk (kg)	146.09 ± 16.39	138.53 ± 29.80	132.87 ± 22.42	137.00 ± 22.12
Trunk /Legs (kg)	174.00 ± 26.56	167.03 ± 42.86	163.30 ± 37.16	162.3 ± 44.65
*Flexibility*				
Sit and reach (cm)	25.65 ± 7.61	24.32 ± 7.64	25.15 ± 6.34	23.10 ± 5.87

* Significant difference between goalkeepers and both defenders and midfielders.

# Significant difference between midfielders and forwards.

## Significant difference between forwards and both midfielders and defenders.

** Significant difference between goalkeepers and all other positions.

$ Significant difference between defenders and midfielders.

## References

[b1-jhk-40-213] Aragón-Vargas LF (2000). Evaluation of four vertical jump tests: Methodology, reliability, validity, and accuracy. Meas Phys Educ Exer Sci.

[b2-jhk-40-213] Arnason A, Sigurdsson SB, Gudmundsson A, Holme I, Engebretsen L, Bahr R (2004). Physical fitness, injuries, and team performance in soccer. Med Sci Sport Exer.

[b3-jhk-40-213] Astrand PO, Rodahl K, Dahl HA, Stromme SB (2003). Textbook of work physiology: physiological bases of exercise.

[b4-jhk-40-213] Ball KA, Best RJ, Wrigley TV (2003). Inter- and inter-individual analysis in elite sport: Pistol shooting. J Appl Biomech.

[b5-jhk-40-213] Borg G, Dahlstrom H (1962). The reliability and validity of a physical work test. Acta Physiol Scand.

[b6-jhk-40-213] Cometti G, Maffiuletti NA, Pousson M, Chatard JC, Maffulli N (2001). Isokinetic strength and anaerobic power of elite, subelite and amateur French soccer players. Int J Sports Med.

[b7-jhk-40-213] Davis JA, Brewer J, Atkin D (1992). Pre-season physiological characteristics of English first and second division soccer players. J Sport Sci.

[b8-jhk-40-213] Essendrop M, Schibye B, Hansen K (2001). Reliability of isometric muscle strength tests for the trunk, hands and shoulders. Int J Ind Ergonom.

[b9-jhk-40-213] Franks AM, Williams AM, Reilly T, Nevill A (1999). Talent identification in elite youth soccer players: Physical and physiological characteristics. Communication to the 4th World Congress on Science and Football, Sydney. J Sport Sci.

[b10-jhk-40-213] Gabbe BJ, Bennell KL, Wajswelner H, Finch CF (2004). Reliability of common lower extremity musculoskeletal screening tests. Phys Ther Sport.

[b11-jhk-40-213] Gil S, Gil J, Ruiz F, Irazusta A, Irazusta J (2007). Physiological and anthropometric characteristics of young soccer players according to their playing position: Relevance for the selection process. J Strength Cond Res.

[b12-jhk-40-213] Hassmen P, Raglin JS, Lundqvist C (2004). Inter-individual variability in state anxiety and self-confidence in elite golfers. J Sport Behav.

[b13-jhk-40-213] Helgerud J, Støren Ø, Hoff J (2010). Are there differences in running economy at different velocities for well-trained distance runners?. Eur J Appl Physiol.

[b14-jhk-40-213] Heller J (2005). Laboratory manual for human and exercise physiology.

[b15-jhk-40-213] Hencken C, White C (2006). Anthropometric assessment of Premiership soccer players in relation to playing position. Eur J Sport Sci.

[b16-jhk-40-213] Hoare DG (2000). Predicting success in junior elite basketball players - the contribution of anthropometic and physiological attributes. J Sci Med Sport.

[b17-jhk-40-213] Inbar O, Bar-Or O, Skinner J (1996). The Wingate anaerobic test.

[b18-jhk-40-213] Leatt P, Shephard RJ, Plyley MJ (1987). Specific muscular development in under-18 soccer players. J Sport Sci.

[b19-jhk-40-213] Lidor R, Côté J, Hackfort D (2009). ISSP position stand: To test or not to test? The use of physical skill tests in talent detection and in early phases of sport development. Int J Sport Exerc Psychol.

[b20-jhk-40-213] Lidor R, Ziv G (2010). Physical and physiological attributes of female volleyball players – A review. J Strength Cond Res.

[b21-jhk-40-213] Lidor R, Ziv G (2011). Physical and physiological attributes of female handball players. Women Sport Physical Act J.

[b22-jhk-40-213] Little T, Williams AG (2007). Measures of exercise intensity during soccer training drills with professional soccer players. J Strength Cond Res.

[b23-jhk-40-213] Malina RM, Chamorro M, Serratosa L, Morate F (2007). TW3 and Fels skeletal ages in elite youth soccer players. Ann Hum Biol.

[b24-jhk-40-213] Morris T (2000). Psychological characteristics and talent identification in soccer. J Sport Sci.

[b25-jhk-40-213] Nikolaidis P, Ziv G, Arnon M, Lidor R (2012). Physical characteristics and physiological attributes of female volleyball players – The need for individual data. J Strength Cond Res.

[b26-jhk-40-213] Parizkova J, Parizkova J, Rogozkin V (1978). Lean body mass and depot fat during autogenesis in humans. Nutrition, physical fitness and health: International series on sport sciences.

[b27-jhk-40-213] Reilly T, Bangsbo J, Franks A (2000a). Anthropometric and physiological predispositions for elite soccer. J Sport Sci.

[b28-jhk-40-213] Reilly T, Williams AM, Nevill A, Franks A (2000b). A multidisciplinary approach to talent identification in soccer. J Sport Sci.

[b29-jhk-40-213] Skinner JS (2005). Exercise testing and exercise prescription for special cases: Theoretical basis and clinical application.

[b30-jhk-40-213] Van Yperen NW (2009). Why some make it and others do not: Identifying psychological factors that predict career success in professional adult soccer. Sport Psychol.

[b31-jhk-40-213] Wong PL, Chamari K, Dellal A, Wisloff U (2009). Relationship between anthropometric and physiological characteristics in youth soccer players. J Strength Cond Res.

[b32-jhk-40-213] Ziv G, Lidor R (2009). Physical attributes, physiological characteristics, on-court performances, and nutritional strategies of female and male basketball players: A review. Sports Med.

[b33-jhk-40-213] Ziv G, Lidor R (2011). Physical characteristics, physiological attributes, and on-field performances of soccer goalkeepers. Int J Sport Physiol Perform.

